# Diagnostic Yield of Ultrasound-guided Trucut Biopsy in Diagnosis of Peripheral Lung Malignancies

**DOI:** 10.7759/cureus.4802

**Published:** 2019-06-02

**Authors:** Rashid A Khan, Vinod Kumar, Muhammad Taimur, Mahrukh A Khan, Mohammad Mohsin Arshad, Mohammad Asim Amjad

**Affiliations:** 1 Pulmonology, Civil Hospital, Jamshoro, PAK; 2 Hospital Medicine, Cleveland Clinic Abu Dhabi, Abu Dhabi, ARE; 3 Internal Medicine, Dow University of Health Sciences, Karachi, PAK; 4 Internal Medicine, Multan Medical and Dental College, Multan, PAK

**Keywords:** pulmonary lesions, ultrasound guided biopsy, lung cancer, histopathology, pakistan, peripheral lung malignancies, sensitivity and specificity

## Abstract

Introduction

While computed tomography (CT) guided lung biopsy has been standard in histological diagnosis of pulmonary lesions, its use is limited to the interventional radiologists only. Ultrasound (US) guided biopsy of pulmonary lesions, which can be performed in-clinic by the pulmonologists only, is becoming a more popular technique. It also has the edge of real-time techniques, multi-planar imaging, and no radiation exposure to the patients.

Methods

This is a retrospective review of all the patients presenting with pleural-based lung lesions who underwent US-guided biopsy for diagnosis in the Department of Pulmonology, Liaquat University of Medical and Health Sciences Hospital, Hyderabad, Pakistan from 1^st^ January 2013 till 31^st^ December 2017. The diagnostic yield, sensitivity, specificity, and accuracy of US-guided biopsies were evaluated for diagnoses of peripheral lung malignancies.

Results

Ultrasound-guided biopsies for lung lesions has a diagnostic yield of 88.3%, sensitivity of 95.80%, and specificity of 90% with an accuracy of 95.35%. Pneumothorax as an immediate complication was seen only in 1.5% cases.

Conclusion

US-guided biopsies are a much safer diagnostic alternative to CT-guided biopsy for lung lesions and have high diagnostic yield. It doesn’t require special radiological interventionists, can be performed at patients' bedsides, and the equipment is not as expensive.

## Introduction

While computed tomography (CT) guided lung biopsy has been standard in histological diagnosis of pulmonary lesions, its use is limited to interventional radiologists only. Ultrasound (US) guided biopsy of pulmonary lesions, which can be performed in-clinic by the pulmonologists only, is becoming a more popular technique. It also has the advantages of real-time techniques, multi-planar imaging, and no radiation exposure to the patients [1]. Ever since screening for lung cancer was introduced, pulmonary nodules were more frequently encountered, were of smaller dimensions, and some were peripherally located. Peripheral pulmonary lesions remain a diagnostic challenge. Sputum cytology and flexible bronchoscopy remain diagnostic modalities for peripheral lesions; however, their yield is low [2]. Fluoroscopy, CT scans, and US-guided biopsy have remained the mainstay for extracting tissue for the diagnosis of peripheral pulmonary lesions [3].

In a study published in India in 2016, the diagnostic yield of ultrasound-guided biopsies was 92.1% with a sensitivity of 92.2% and specificity of 91.7% [1]. Ultrasound-guided biopsy provides a complete view of lung masses for biopsy. However, this technique is still underutilized despite its high diagnostic yield, real-time guidance, and the benefits of being performed at bedsides without any radiation exposure [4-10].

To our best knowledge, the only local study available, from central Pakistan, to assess the efficacy of US-guided trucut biopsy reveals overall diagnostic yield to be 98% with a 98% sensitivity [11]. US-guided techniques can be very beneficial in a low-resource country like Pakistan. It reduces the time and cost of diagnosis. It is an easy-to-perform technique, provided experienced pulmonologists are available. The aim of this study is to assess the diagnostic yield, sensitivity, and specificity of US-guided trucut biopsies in peripheral malignant lung lesions.

## Materials and methods

In this retrospective analysis, all medical records of all patients presenting with peripheral lung lesions who underwent US-guided biopsy for diagnosis in the department of pulmonology at Liaquat University of Medical and Health Science Hospital, Hyderabad, Pakistan from 1^st^ January 2013 till 31^st^ December 2017 were included. Patient age and gender were included. Histological diagnosis on US-guided biopsy was included.

For US guidance, Micro Maxx (Sonosite Inc., Bothell, WA, USA) unit equipped with a 1-5 MHz phased array transducer was used. Patients were placed in a comfortable position depending on the location of the lesion. Lesion location was achieved by scanning the intercostal spaces, and Doppler scan was used to bypass the vessels from the biopsy path. Biopsy site was then disinfected (2% chlorhexidine, 70% isopropyl alcohol) and local anesthesia (2% lidocaine hydrochloride injection) was given. The biopsy was performed using a trucut needle under real-time guidance with US. The biopsy sample was saved in a formalin jar and sent for histopathology.

Diagnostic yield, sensitivity, specificity, and accuracy of the procedure was evaluated using binary classification [12]. Post-procedure US was done with suspicion of iatrogenic pneumothorax. Patients were monitored in the recovery room for an hour where chest radiography was conducted.

## Results

During the study period, 129 patients underwent US-guided lung biopsy for diagnosis of peripheral lung malignancies. Out of these, 106 (84.1%) were male and 20 (15.9%) were females. Their mean age was 69 ± 9 years. The diagnostic yield of ultrasound-guided biopsies was 88.3% (114/129). The most common histological type was squamous cell carcinoma (63.72%) followed by small-cell lung carcinoma (28.57%) as seen in Table 1. 

**Table 1:** Histological Diagnoses of Ultrasound-guided Lung Biopsy (n=114)

Histological Diagnosis	Frequency (%)
Adenocarcinoma	4 (3.5%)
Atypical cell	2 (1.8%)
Bronchioloalveolar mucinous-type carcinoma	1 (0.8%)
Large-cell carcinoma	1 (0.8%)
Small-cell lung carcinoma	32 (28.1%)
Squamous cell carcinoma	72 (63.2%)
Undifferentiated (anaplastic) carcinoma	2 (1.7%)

There was one (0.7%) false positive case; diagnosed as squamous cell carcinoma which turned out to be an abscess in surgical resection. There were five (3.9%) false negative cases of which three were diagnosed with repeat ultrasounds and two were diagnosed with CT-guided biopsy. There were nine (6.9%) cases of true negative, of which six of them had anthracosis, two had abscesses, and one had atypical cell. The specificity and sensitivity of US-guided trucut biopsy in our sample is shown in Table 2. There were two cases (1.5%) of pneumothorax in immediate post-ultrasound guided biopsy.

**Table 2:** Binary Classification Tests of Ultrasound-Guided Trucut Biopsy

Binary Classification Tests	Value %
Sensitivity	95.80% (90.47% to 98.62%)
Specificity	90.00 % (55.50% to 99.75%)
Positive Predictive Value	99.13% (94.67% to 99.86%)
Negative Predictive Value	64.29 % (42.69% to 81.31%)
Accuracy	95.35% (90.15% to 98.27%)

## Discussion

This study is the only one of its type as far as data from Pakistani population is concerned. It has shown a yield of around 90% with US-guided biopsy in pleural-based pulmonary lesions. This study has shown US-guided biopsy to be an accurate diagnostic modality, with a yield of 88.3%, with lesser harmful effects. US-guided techniques have shown high sensitivity, specificity, accuracy, and positive predictive values that help with the diagnosis of pulmonary malignancies. These results are also comparable to the results of other studies [1, 4, 8, 10-11].

Several limitations of our study should be considered. This retrospective review could not exclude patient selection bias. There is no knowledge of the number of needle punctures attempted, and time required for the procedure. This data is from a tertiary care public center where most of the malignancies and complicated cases are referred, hence, the higher frequency of cancer diagnosis. This frequency cannot be generalized and multicentre studies are essential for this purpose.

One of the major problems with CT guided biopsy is pneumothorax [13-15]. In our study, only two (1.5%) patients had an incidence of pneumothorax, which is comparable to other studies found in the literature [1, 4, 10, 13]. Sconfienza et al. suggested that fewer complications with US-guided biopsy as compared to CT-guided biopsy are attributed to real-time guidance which helps in accurate needle access and reduces the incidence of multiple punctures [10]. Along with real-time guidance, US-guided procedures do not expose the patients to radiations. However, patients utilizing CT-guided biopsy as a diagnostic modality are exposed to harmful radiation. Although, the cost difference was not analyzed in this study; the machinery for ultrasound technology is less expensive than a CT scanner, hence making it the most readily available diagnostic modality for most low-resource centers [16-17]. For real-time guidance, there is still CT fluoroscopy, but it only gives transverse sections and then the problem with radiation exposure sustains subject's patients to high radiation exposure [3]. US machines are portable and small in size, they can be taken to the patient's bedside instead of patient being mobilized. They also allow the procedure to be performed in whichever position the patient is comfortable in [1].

This study has its shortcomings too. First, it was a retrospective analysis which brings in patient selection and recall bias. Second, only patients undergoing US-guided biopsy were included. No comparison was done with patients undergoing CT-guided biopsy. We also do not have records of the number of patients rendered unsuitable for US-guided biopsy. There was also no record of the number of needle punctures attempted in each patient and the duration of the procedure. These were not documented in patient records. This study is from a large center where most of the malignancies are referred for management. This center has the expertise of experienced pulmonologists to perform these procedures. Hence, the results cannot be generalized for all centers.

## Conclusions

In conclusion, US-guided biopsy is a safe procedure with high diagnostic yield. It doesn’t require special radiological interventionists and can be performed at the patients' bedsides by experienced pulmonologists. It does not expose the patients to unnecessary radiations. The machinery is not as expensive and easily accessible, especially in countries like Pakistan, where cost is a major drawback. Larger comparative trials must be conducted to generalize these conclusions.
